# Formamidinium Lead Iodide Perovskite Films with Polyvinylpyrrolidone Additive for Active Layer in Perovskite Solar Cells, Enhanced Stability and Electrical Conductivity

**DOI:** 10.3390/ma14164594

**Published:** 2021-08-16

**Authors:** Vedran Kojić, Mario Bohač, Arijeta Bafti, Luka Pavić, Krešimir Salamon, Tihana Čižmar, Davor Gracin, Krunoslav Juraić, Mirela Leskovac, Ivana Capan, Andreja Gajović

**Affiliations:** 1 Ruđer Bošković Institute, Bijenička cesta 54, HR-10000 Zagreb, Croatia; vkojic@irb.hr (V.K.); mario.bohac@irb.hr (M.B.); luka.pavic@irb.hr (L.P.); Kresimir.Salamon@irb.hr (K.S.); tihana.cizmar@irb.hr (T.Č.); davor.gracin@irb.hr (D.G.); krunoslav.juraic@irb.hr (K.J.); ivana.capan@irb.hr (I.C.); 2 Faculty of Chemical Engineering and Technology, University of Zagreb, Marulićev trg 19, HR-10000 Zagreb, Croatia; abafti@fkit.hr (A.B.); mlesko@fkit.hr (M.L.)

**Keywords:** formamidinium lead iodide perovskite, polyvinylpyrrolidone, ZnO nanorods, optical properties, electrical properties

## Abstract

In this paper, we studied the influence of polyvinylpyrrolidone (PVP) as a stabilization additive on optical and electrical properties of perovskite formamidinium lead iodide (FAPI) polycrystalline thin films on ZnO nanorods (ZNR). FAPI (as an active layer) was deposited from a single solution on ZNR (low temperature processed electron transport layer) using a one-step method with the inclusion of an anti-solvent. The role of PVP in the formation of the active layer was investigated by scanning electron microscopy and contact angle measurements to observe the effect on morphology, while X-ray diffraction was used as a method to study the stability of the film in an ambient environment. The effect of the PVP additive on the optical and electrical properties of the perovskite thin films was studied via photoluminescence, UV-Vis measurements, and electrical impedance spectroscopy. We have demonstrated that PVP inclusion in solution-processed perovskite FAPI thin films prevents the degradation of the film in an ambient atmosphere after aging for 2 months. The inclusion of the PVP also improves the infiltration of FAPI perovskite into ZnO nanostructures, increases electrical conductivity and radiative recombination of the photo-generated charge carriers. These results show promising information for promoting PVP stabilized FAPI perovskites for the new generation of photovoltaic devices.

## 1. Introduction

After the first report of a unique photoactive material based on lead halide compounds by Kojima et al. [1], a whole new field of scientific research regarding inorganic-organic lead halide perovskites (LHP) emerged. LHP employs an ABX_3_ crystal structure, where lead acts as a divalent cation (B) with halide elements (I^−^, Br^−^) serving as anions (X), while methylammonium (MA^+^), formamidinium (FA^+^), or caesium (Cs^+^) as monovalent cations (A). A large number of published articles have studied the MA variant perovskites [2]. In contrast to MA perovskites, the larger FA cation ensures that the Goldschmidt tolerance factor is brought closer to the expected value of 1.0 [3], thus favoring the formation of the photoactive cubic perovskite. FAPbI_3_, usually referred to as FAPI, perovskite bandgap gives a measured value of 1.43 eV [4], which is closer to the ideal bandgap value according to the Shockley–Queisser limit curve [5] than the value of measured MAPbI_3_ counterpart of 1.51 eV. On the other hand, FA-based perovskites have an unstable phase structure: under ambient and humid atmosphere the perovskite cubic α-FAPI transforms into the undesirable non-perovskite yellow δ-FAPI polymorph [6]. In order to improve the phase stability of the cubic α-FAPI, researchers have attempted various approaches: doping FAPI with MA [7,8,9,10,11] or Cs [12,13,14,15] cations, introducing chloride anions into the perovskite structure [16], forming perovskites of lower dimensionality [17,18], introducing additives (as thiourea [19] or N-methyl-2-pyrrolidone [20]) into the perovskite solution, or the attempt on interfacial modification [21,22] of the electron transport layers (ETL).

Recently, several groups reported stabilization of the cubic perovskite phase in mixed cation perovskites or all-inorganic perovskites by the use of polymer polyvinylpyrrolidone [23,24,25,26]. Yan et al. [23] reported that adding a concentration of 1.33 mg/mL of PVP improved the film quality and cell performance by using a mixed cation MA_x_FA_1–x_PbI_x_Br_1–x_ perovskite as the active layer and SnO_2_ as the ETL. They have shown how PVP forms a stable complex MA/FAX-PbX_2_-PVP (X = I^−^, Br^−^). This complex can improve the stability of the δ-phase, which leads to the slower crystallization of the α-phase and forms films with fewer traps. PL signal was increased with the addition of PVP, while electrochemical impedance spectroscopy showed decreased charge recombination in the active layer. Xiong et al. [24] also studied a mixed cation perovskite (MA_x_FA_1−x_PbI_x_Br_1−x_) on SnO_2_ ETL by introducing the concentration of 80 mg/mL of PVP into the perovskite solution which led to films protected from degradation under humid environment as well as improved mechanical flexibility. Veeramuhutu et al. [25] showed that the addition of 50 mg/mL of PVP enhanced the radiative recombination processes in the all-inorganic CsPbBr_3_ perovskite layer as well as reducing surface defects. They combined PVP for both the active layer and the interlayer between the HTL and the active layer. Their device architecture was inverted with 2,2′,2″-(1,3,5-Benzinetriyl)-tris(1-phenyl-1-H-benzimidazole), or TPBi, as an ETL. Li et al. [26] also combined PVP, at a concentration of 100 mg/mL, with the all-inorganic perovskite layer CsPbI_3_ on TiO_2_ and reported a nearly double increase in carrier diffusion length. They also reported that chemical bonding between the acylamino group in PVP and CsPbI_3_ enhances the electron cloud density which leads to reduced surface tension.

Moreover, Zhou et al. [27] used PVP as an interlayer between PCBM electron transport layer (ETL) and Ag cathode in the inverted planar heterojunction perovskite solar cells with MAPI active layer, and obtained efficiency enhancement by 15.9% relative to that of the device without PVP interlayer. On the other hand, Di et al. [28] applied mixtures of PVP and polyvinyl alcohol to stabilize mixed cation perovskite solar cells using ZnO nanorods covered with ZnSe as ETL. The enhancement of the efficiency and air stability of PSCs is observed even after 30 days (over 82.2% of initial efficiency is retained in an atmospheric environment with 70% humidity). The same group of authors [29] also studied the incorporation of the same mixture of PVP/polyvinyl alcohol for the stabilization of MAPI perovskite in PSC with ZnO@TiO_2_ nanorod arrays as ETL. They showed better air stability of PSC compared to the device with single polymer or without polymer, while photoelectric conversion efficiency of the PVP/PEG-modified device was 30% better than that of the unmodified one. The authors explained this observation by the better quality of perovskite film due to the formation of the chemical bonds between the polymers and the perovskite.

**Figure 1:**
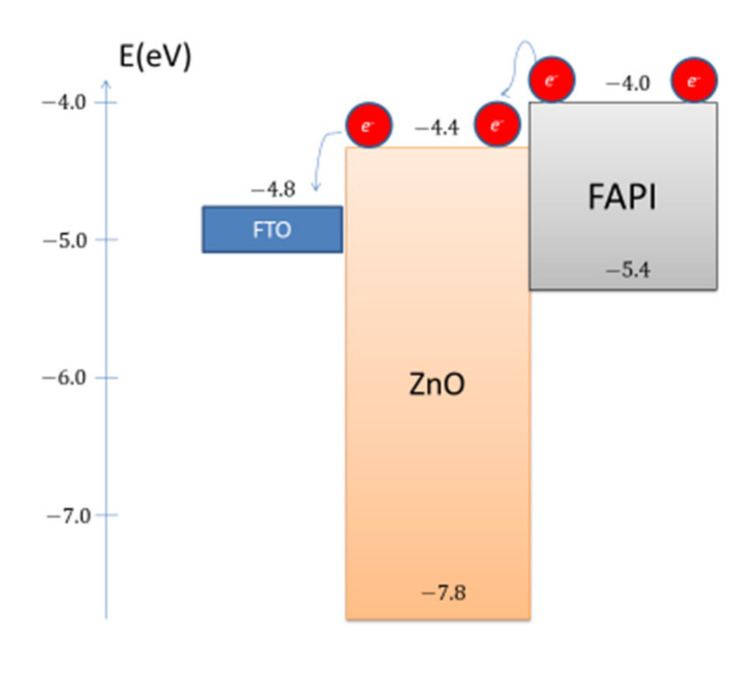
A scheme for energy diagram of ZnO/FAPI heterojunction.

Although the composition of the perovskite active layer has a major role in the stability and properties of PSC, the material for the ETL layer is also responsible for the stabilization of perovskite material. Therefore, instead of standard TiO_2_, authors use SnO_2_ [30] and ZnO [31] materials for ETL. The role of ZnO in perovskite devices is the separation and transport of charges, specifically electrons, from the perovskite material towards the cathode. With its bandgap of 3.4 eV and lower chemical potential of ZnO on the absolute scale [32], a heterojunction contact drives the charge separation (Figure 1). With higher electron mobility and lower processing temperatures, ZnO is a more practical material in comparison to its counterpart TiO_2_ in its role as a solar cell ETL [33].

Nanostructured ZnO nanorods as ETL were first reported by Son and colleagues [34] by using MA-based perovskite as the active layer. While achieving a highly efficient device with a power conversion efficiency (PCE) of over 11%, they reported incomplete pore filling of the ZnO with the perovskite. A year later, the same group [35] reported the influence of seed layer growth of ZnO nanorods on the perovskite performance. They found that the surface modification of ZnO nanorods can suppress the charge carrier recombination at the ETL/perovskite interface. On the other hand, Bi et al. [36] reported fast recombination rates at the ZnO/perovskite interface, which is more evident with the increase in nanorod length. Yang et al. [37] discussed the thermal instability of MA-based perovskites on ZnO. In short, the basic nature of the ZnO surface (isoelectric point >8.7) deprotonates the methylammonium cation (pKa value of 10.6), facilitates the thermal decomposition, indicating that the source of the degradation lies in the ZnO/perovskite interface. Song et al. [38] fabricated FAPI perovskite on ZnO nanoparticles via the two-step solution dipping method. In their work, they have compared FAPI stability with the MAPI perovskite and report that FAPI does not degrade on ZnO ETL materials.

In this work, we have investigated the stabilization of pure FAPI perovskite films by incorporation of PVP as an additive using grazing incidence XRD. The study of PVP on pure FAPI perovskite excludes the influence of multi-cation stabilization, applied by other authors. The influence of PVP on morphology was studied by SEM, while optical and electrical properties were studied by PL, UV-Vis spectroscopy, and impedance spectroscopy, respectively. The influence on morphology and stability was observed by comparing- the PVP-infused and pure perovskite FAPI thin films deposited on ZnO nanorods as ETL. Optical measurements were made by comparing FAPI perovskite films with and without PVP on both glass substrates and nanostructured ZnO thin film substrates. The influence of PVP on the conductivity of the FAPI films deposited on a nanostructured ZnO ETL was studied at various temperatures. With the acquired electrical data, we determined corresponding activation energies in the PVP-infused and pure perovskite thin films under an inert (nitrogen) atmosphere. In addition, we report a positive effect of PVP on photoconductivity.

## 2. Materials and Methods

### 2.1. Materials and Preparation Procedures

For the synthesis of the formamidinium based perovskites, the following materials were used: lead (II) iodide (Sigma-Aldrich, 99%, St. Louis, Mo, USA), formamidinium iodide (FAI) (Sigma-Aldrich, ≥99%), N,N-Dimethylformamide (DMF) (Merck, p.a., Darmstadt, Germany), dimethyl sulfoxide (DMSO) (Merck, p.a.), Chlorobenzene (Merck, p.a.), polyvinylpyrrolidone (PVP) (Alfa Aesar, M.W. 40 000, Kandel, Germany). ZnO nanostructures for ETL were prepared using: zinc diacetate dihydrate (Fluka, ≥99%, Seelze, Germany), ethanolamine (Sigma–Aldrich, ≥99.5%), zinc nitrate hexahydrate (Acros, 98%, Geel, Belgium), hexamethylenetetraamine (Sigma–Aldrich, ≥99.5%), while fluorine-doped tin oxide (FTO) substrates (Sigma-Aldrich, ρ ~ 7 Ω/sq) were used as conductive glass electrodes.

In order to obtain the perovskite precursor solution, PbI_2_ and FAI were dissolved in an organic solvent mixture containing DMF and DMSO (*V*(DMF):*V*(DMSO) = 4:1) in the concentration of 1 mmol/mL. For the precursor solution containing the additive, PVP was dissolved to form an 8 mg/mL solution. This concentration was chosen for the study after optimization of the prepared films due to the long-term stability. The perovskite films were prepared in one step with the addition of the anti-solvent (chlorobenzene). Other authors report various ranges of PVP concentrations in perovskite thin layers, from 1.1 mg/mL to 100 mg/mL [23,24,25,26], but their introduction of PVP in the systems is diverse from our simple FAPI material.

ZNR thin films were prepared in a two-step process on FTO glass substrates, based on the preparation routes described in Foo et al. [39] and Panžić et al. [40]. Prior to ZNR preparation, the substrates were ultrasonically cleaned for 10 min in acetone and isopropanol, respectively, to be later rinsed in ethanol, water and then dried in an N_2_ stream. The first step of ZNR preparation includes spin coating (2000 rpm for 20 s) 70 µL of a seed layer solution consisting of 0.25 mmol/mL zinc acetate dihydrate dissolved in ethanol with an equimolar addition of ethanolamine. The substrates with the spin-coated seed layers are then dried on a hotplate at 150 °C for 15 min and later annealed in air at 350 °C for 2 h in a tube furnace. This results in a crystalline ZnO seed layer thin film. The second step includes nanorod growth in which the crystalline seed layers are put into a ZNR growth solution, containing 35 μmol/mL zinc nitrate hexahydrate and 35 μmol/mL hexamethylenetetramine, upside down using substrate holders and kept at 90 °C for 2 h. Afterwards, the samples were removed from the growth solution, rinsed in water, dried in an N_2_ stream, and annealed in air at 450 °C for 2 h.

Both the perovskite films on glass substrates and on ZNR substrates were prepared inside a nitrogen-filled glovebox by spin coating 50 µL of the perovskite precursor. The spin coating parameters were: 1000 rpm for 10 s and 6000 rpm for 20 s. During the last 10 s, 50 µL of chlorobenzene were spun-coated to improve the nucleation and crystal growth of the film. The prepared substrates were annealed on a hotplate at 200 °C for 1 min.

### 2.2. Methods of Characterization

The crystalline structure of the films was studied by X-ray diffraction analysis (XRD) using the Siemens D5000 diffractometer (Dresden, Germany) equipped with Cu anode, Goebel mirror, and graphite monochromator in front of the point detector. The experiments were performed in the grazing incidence geometry with a nominal incidence angle of 1.3° with respect to the film surface plane. Such conditions guaranteed that the whole PVP-FAPI film was irradiated, despite the possible reduction of the incident angle due to the unwanted (small) inclination of the sample at the sample stage.

SEM was measured with field emission SEM, model JSM-7000F manufactured by JEOL Ltd. (Tokyo. Japan), using an accelerating voltage of 5 kV.

The surface tension of perovskite solutions with and without PVP was measured by the pendant drop method using a DataPhysics OCA 20 Instruments GmbH goniometer. The measurements were carried out at a temperature of (23.0 ± 0.2) °C. The analysis was carried out using the SCA 22 software module, which allows the measurement of surface and interfacial tensions using the pendant drop method. The average values of at least five drops were taken and the standard deviation was less than 1°.

The contact angles were measured by the sessile drop method by placing a drop of the DMF/DMSO and PVP-DMF/DMSO solutions on both the ZnO seed layer and a glass plate. The film of the formed droplet was captured by the CCD camera of OCA and the contact angle was measured 1–2 s after the formation of the droplet. The average values of at least five droplets on different sites of the same sample were taken and the standard deviation was less than 1°.

Optical properties were studied by UV-Vis spectroscopy and photoluminescence spectroscopy (PL). UV-Vis measurements were performed using an Ocean Optics HR4000 spectrometer (Ocean Optics, Largo, FL, USA) equipped with an integration sphere and a Xe 150 W light source. Photoluminescence (PL) spectra were recorded using a LDM405 405 nm laser diode module (Thorlabs, Newton, NJ, USA) as an excitation source. The emitted PL light was analyzed by a BRC112E CCD array spectrometer (B&W Tek, Newark, NJ, USA).

The electrical conductivity of prepared perovskite thin films on ZNR/FTO-coated glass substrate was measured by impedance spectroscopy (Novocontrol Alpha-A Dielectric Spectrometer, Novocontrol Technologies, Montabaur, Germany) in the frequency range from 0.1 Hz to 1 MHz at a voltage of 50 mV in a nitrogen atmosphere at temperatures between 30 and 100 °C in heating (1st run) and subsequent cooling (2nd run). A frequency sweep at each temperature (step 10 °C) was repeated twice. For electrical contacts, platinum wires were attached on the surface of FTO glass substrate and perovskite thin films thus enabling electrical characterization of the film in the cross-sectional geometry. Photoconductivity measurements were performed at 1 Hz under the illumination of 1 Sun in the ambient atmosphere using the same contact setup. In order to check the reliability of the results, for both thin-film samples, the measurements were performed three times (each time on different sample areas) under identical experimental conditions.

## 3. Results and Discussion

### 3.1. Structure and Morphology

#### 3.1.1. X-ray Diffraction

**Figure 2:**
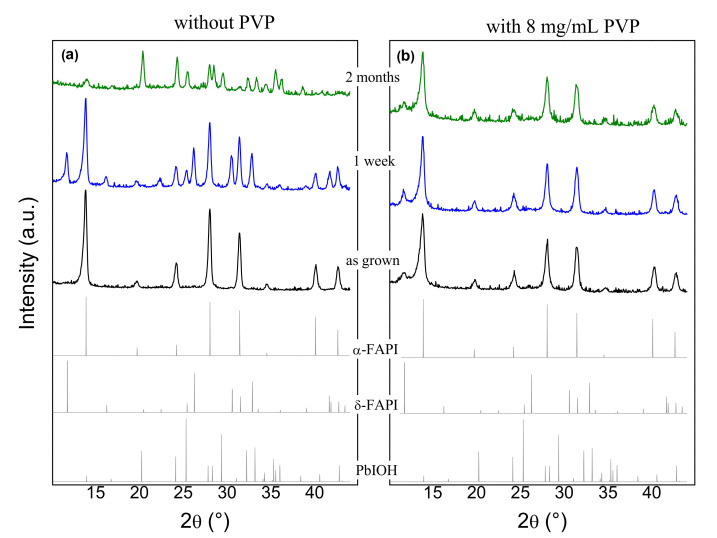
Time dependence XRD patterns of thin films on a glass substrate: (**a**) pure FAPI film, (**b**) FAPI film containing 8 mg/mL PVP.

In order to evaluate the impact of the PVP additive as a stabilizing agent for perovskite materials, we first studied the structural properties of the films prepared on bare glass substrates. Figure 2 represents XRD patterns of films without PVP and with 8 mg/mL PVP. The age of the film when XRD measurements were performed is indicated next to the corresponding pattern. At the bottom of Figure 2 calculated reference powder diffractograms for the α-FAPI, δ-FAPI, and PbIOH phases are shown.

In the case of pure FAPI thin film without PVP, the as-grown film reveals a pure cubic α-FAPI phase (Figure 2a). The perovskite phase gradually degrades under ambient conditions (outside of the nitrogen-filled glovebox). After one week, the α-FAPI phase partially transformed into the non-perovskite δ-FAPI (yellow) phase, and then completely into the pure Pb(OH)I (pale white) phase. The formation of the Pb(OH)I phase, i.e., inclusion of hydroxide anions into the structure, confirms the reaction of the perovskite crystals with air moisture under the influence of the ambient conditions. The α-FAPI phase dominated in the 8 mg/mL PVP-infused as-grown film (Figure 2b), but here a weak diffraction peak at 11.8° of δ-FAPI phase (non-perovskite) can be seen. In contrast to the FAPI thin film without PVP, the film with PVP shows long-term structural stability. This indicates that PVP prevents the alpha phase degradation to non-perovskite δ polymorph. The main mechanism behind this is not completely clear, but it could be inferred that PVP resides at the grain boundaries, and thus reduces the area where moisture-induced degradation can occur [41]. In addition, a small fraction of δ-FAPI found in the as-grown 8 mg/mL PVP-infused film, indicates that the chemical interaction of PVP with δ-crystallites increases activation energy for the phase transition into α-phase during the syntheses procedure. Similar to that, a mechanism based on the FAPI-PVP interaction can be relevant for the stabilization of α-crystallites [23].

**Figure 3:**
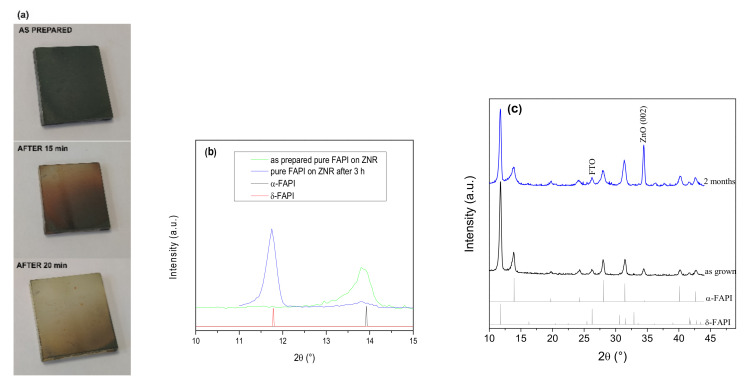
(**a**) Photographs of rapid degradation of pure FAPI film (α-phase is black, δ-phase is yellow), (**b**) low-angle XRD patterns of the pure FAPI thin film on ZnR and (**c**) time-dependent XRD patterns of the FAPI thin-film infused with PVP on ZNR. A higher intensity of the ZnO (002) peak in the pattern of the aged sample is a consequence of the larger higher incidence angle of X-rays which resulted in a larger penetration depth.

We further investigated the stability of the FAPI perovskite phase grown on ZnO nanorods (ZNR) as such structure is intended for use in photovoltaic cells with nanostructured ZnO as an electron transport layer. In the case of FAPI film without PVP on ZNR, we found rapid degradation of the pure α-FAPI phase into δ polymorph under the ambient conditions (Figure 3a,b). This is not consistent with the findings of Song et al. [38], where it was reported that FAPI films are chemically stable on the basic ZnO surface. When adding PVP, as already shown for the same film grown on a glass substrate (Figure 2b), perovskite α-phase grown on ZNR is structurally stable (see Figure 3c). Furthermore, the as-grown film with PVP on ZNR contains δ-phase, similar to the films on the bare glass substrate. Here, a strong intensity of δ-(100) diffraction peak at 11.8° might suggest a higher fraction of δ-phase present for the case of ZNR substrate than for the bare glass substrate. However, the δ-(100) diffraction peak is the only visible diffraction peak for the δ-phase, indicating highly oriented δ-grains. Presumably, highly oriented ZNR substrate ((001) texture) influences the (100) texture of initially developed δ crystallites before annealing. Contrary to that, the α-phase, transformed from the δ-phase during the annealing process, shows all diffraction peaks (see Figure 2and Figure 3b), revealing randomly oriented α-crystallites. Taking highly oriented δ-grains and randomly oriented α-crystallites into account, the peak intensity ratio of α- and δ-phase observed on film growth on ZNR, corresponds to the similar ratio of these two phases as for the bare glass substrate.

#### 3.1.2. Scanning Electron Microscopy (SEM)

**Figure 4:**
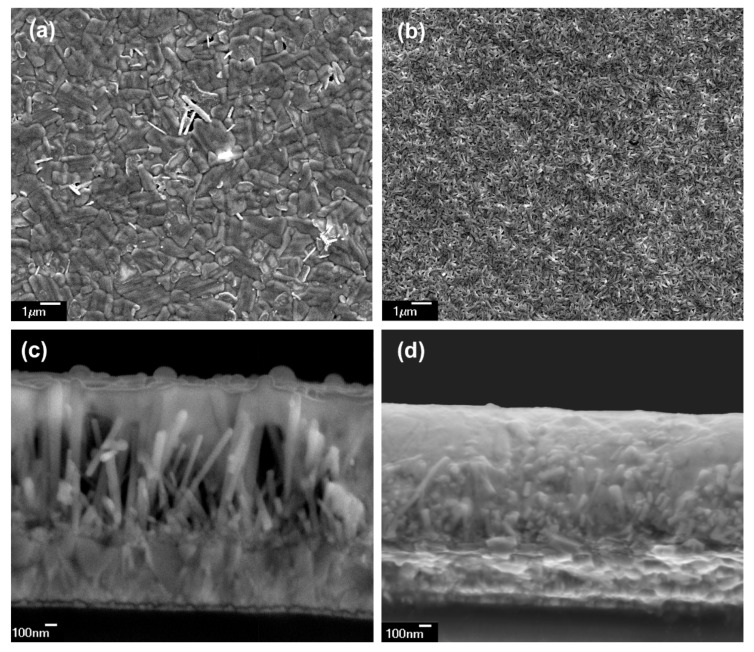
SEM micrographs: (**a**) top view of FAPI perovskite on ZNR, (**b**) top view of FAPI perovskite with PVP on ZNR, (**c**) cross-sections of FAPI perovskite on ZNR, and (**d**) cross-sections of FAPI perovskite with PVP on ZNR.

In order to study the morphology of the surface and infiltration of the formed FAPI layer on ZNR, SEM measurements were conducted (Figure 4). The top view of the FAPI perovskite layer without PVP shows a nonhomogeneous surface with some macroscopic defects indicating separation of phases (Figure 4a). Although, the observed grains are larger than in the case of PVP-infused FAPI (Figure 4b), their shape is irregular and an additional phase can be seen at the grain boundaries. On the other hand, PVP-infused FAPI shows a homogenous surface with small regularly shaped grains (Figure 4b). It seems that the formation of the smaller grains supported the infiltration of the perovskite between ZNR as can be seen in the cross-section images (Figure 4c,d).

The cross-section SEM indicates only surface attachment of pure FAPI perovskite film without PVP, with incorporation only between some ZNR (Figure 4c). On the other hand, complete infiltration between ZNR of FAPI perovskite infused with PVP was observed (Figure 4d). It could be seen that the complete layer of FAPI on ZNR was thinner in the case of PVP-infused FAPI. A thinner layer of PVP-infused FAPI is expected since the same volume of perovskite solution was used during the preparation of FAPI without PVP and FAPI with PVP. FAPI with PVP infiltrated between ZNR, thus reducing the total thickness of the layers. We interpret the incorporation of FAPI with PVP as the consequence of the polarity of the solution used for the preparation of the perovskite layer and surface energy of the ZNR, as will be explained in the next section. Moreover, since pure FAPI perovskite was not completely incorporated between ZnO nanorods, the space between ZNR in the structure enabled moisture from the environment to interact with FAPI which additionally contributed to the degradation of the non-stabilized FAPI.

### 3.2. Surface Tension and Contact Angle

To explain the difference in the infiltration of pure FAPI perovskite and FAPI with PVP into the ZnO nanostructures, observed from the SEM images, surface tension (ST), and contact angle measurements were performed. By measuring the ST of the solvent, PVP/solvent mixture, and comparing their values, we can investigate the impact of the polymer on the molecular interactions inside the solvent. By dissolving large polar macromolecules in organic solvents, we enhanced polar interactions inside the system. The PVP polymer contains both a polar pyrrolidone ring and a carbonyl group which can interact with polar groups from both DMF and DMSO and, by extension, have the consequence of influencing the solution-substrate contact. By measuring the ST of the solvent and PVP/solvent solution via the pendant drop shape analysis, we have calculated the following average values: *ST*_avg_(solvent) = (37.51 ± 0.55) mN/m and *ST*_avg_(PVP/solvent) = (38.86 ± 0.19) mN/m. Although a small increase in surface tension in the PVP/solvent case was observed, it can be explained with the aforementioned polar interaction increase propagated with the PVP inclusion.

**Figure 5:**
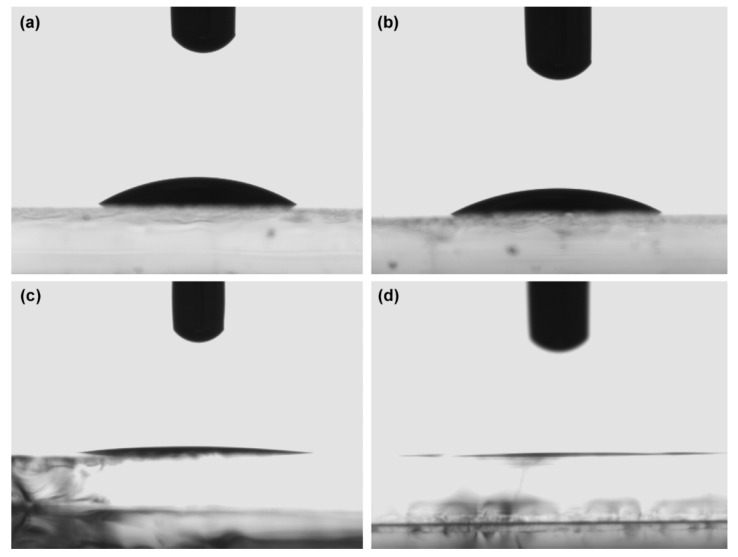
Contact angles of: (**a**) DMF/DMSO on glass, (**b**) PVP-DMF/DMSO on glass, (**c**) DMF/DMSO on ZnO seed layer, and (**d**) PVP-DMF/DMSO on ZnO seed layer.

To examine the solution spreading effect, we have measured the contact angle (CA) for both the solvent and PVP/solvent mixture on glass and ZnO seed layer substrates (Figure 5).

Table 1 shows the contact angle values of DMF/DMSO and the mixture of PVP with DMF/DMSO. The data confirms that the ZnO seed layer has higher total surface energy in comparison to glass substrates. The lower contact angles show how droplet spreading is more pronounced on surfaces with higher surface energies. By infusing the solvent with a material that enhances the polar interactions, in our case PVP, the increased polar component of the liquid results in a larger spreading of the droplet [42]. We can conclude that PVP promotes the increase in droplet spreading in both glass and ZnO surfaces and, by extension, better wetting of the solution upon the substrate.

### 3.3. Optical Properties

#### 3.3.1. Photoluminescence Measurements

**Figure 6:**
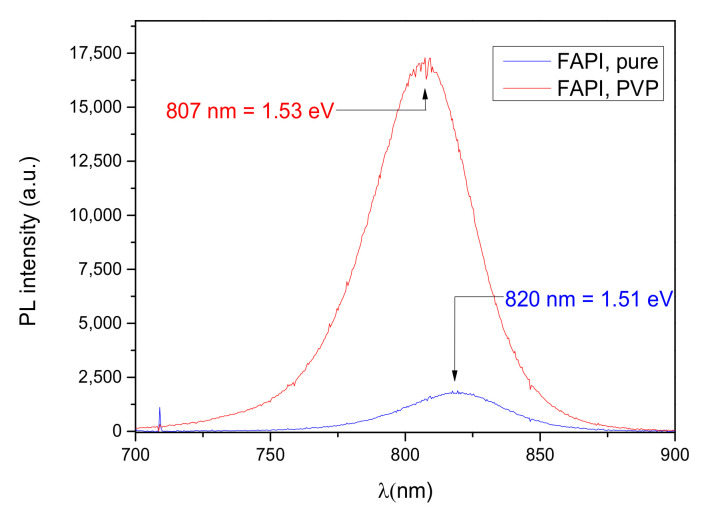
Photoluminescence spectra of FAPI thin film (pure) and FAPI thin film infused with PVP on glass substrate.

Once we have confirmed the structural stability of the material, we have examined the optical properties of the perovskite with and without the PVP additive, as well as their variability in a given time period. Figure 6 reports a significant PL intensity increase with the addition of PVP. This increase is comparable with the reported increase in the mixed cation perovskite variant and the Cs cation variant [23,25]. In those reports the increase is attributed to the elimination of the dangling Pb^2+^ bonds which are stabilized by the carbonyl group of the PVP, decreasing the surface defects and suppressing non-radiative recombination. With the PL intensity increase, we observed a blue shift in the recombination wavelength when PVP was added into the thin film (λ_FAPI_, pure = 818 nm, λ_FAPI,PVP_ = 806 nm).

**Figure 7:**
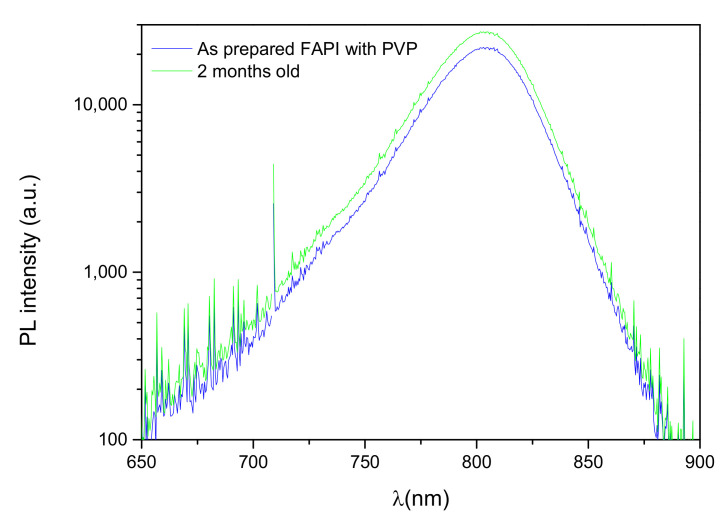
Photoluminescence spectra of FAPI thin film infused with PVP on ZNR: as prepared (blue) and after aging in ambient conditions for 2 months (green).

To support the claim that PVP-infused FAPI films stability is greatly enhanced on nanostructured ZnO substrates, Figure 7 shows that the PL intensity was similar in the as-prepared sample and in the sample recorded after 2 months of aging in ambient conditions. This result suggests that aging under ambient conditions does not impact the crystal grain boundaries nor increase surface defects.

#### 3.3.2. UV-Vis Measurements

**Figure 8:**
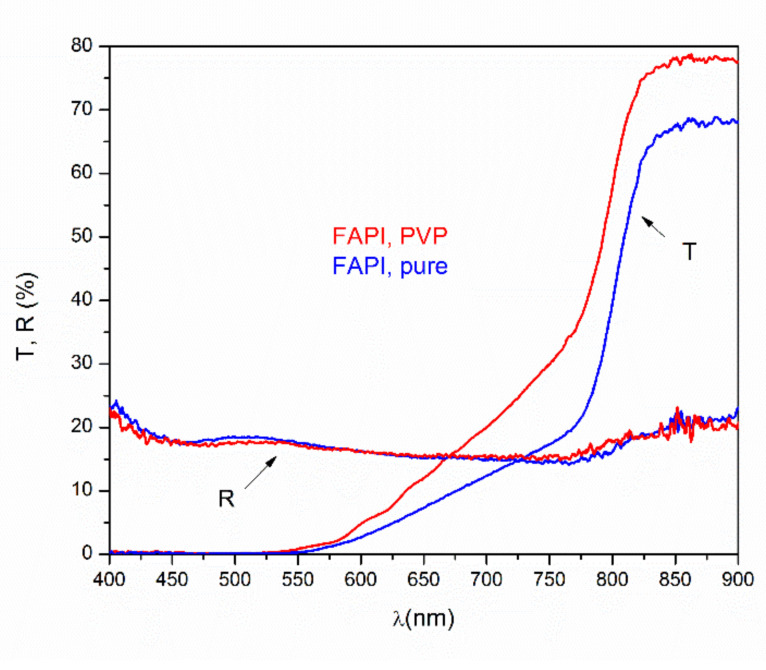
Transmittance and reflectance of FAPI perovskite with and without PVP.

**Figure 9:**
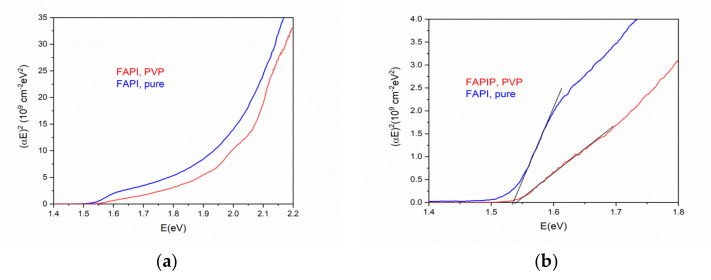
(**a**) The plots of (*αE*)^2^ vs photon energy E for FAPI perovskite with and without PVP (**b**) zoomed region of the Tauc plot used for the bandgap *E_g_* estimation.

The transmittance and reflectance of the FAPI thin film without PVP (pure) and the FAPI with PVP deposited on glass substrate are plotted in Figure 8. The transmittance of the sample with the addition of PVP is larger than pure FAPI, indicating a lower absorption coefficient since the thickness and the reflectivity of both samples are the same. Absorption coefficients were estimated from the following expression [43] that is strictly valid for the self-standing film:

(1) $$T={\left(1-R\right)}^{2}\times e^{-\alpha d}$$

where *T*, *R*, *α*, and *d* are transmittance, reflectance, absorption coefficient, and the thickness of the samples, respectively. The effect of multiple reflections on the interfaces is not so pronounced due to surface roughness and non-uniformity of density and thickness of deposited films. Therefore, the analysis by using Equation (1) provides useful data for comparison between FAPI with and without the addition of PVP. The calculated absorption coefficient was used for the Tauc plot (Figure 9a) from which an optical bandgap, *E_g_*, was estimated (Figure 9b), assuming direct optical transition and the equation [44]:

(2) $${\left(\alpha E\right)}^{2}=A\times \left(E-Eg\right)$$

in the narrow interval between photon energies 1.5 and 1.6 eV. The values for *E_g_* obtained in this way are 1.53 eV for pure FAPI and 1.54 eV for FAPI with PVP, which is in agreement with the photoluminescence measurements.

**Figure 10:**
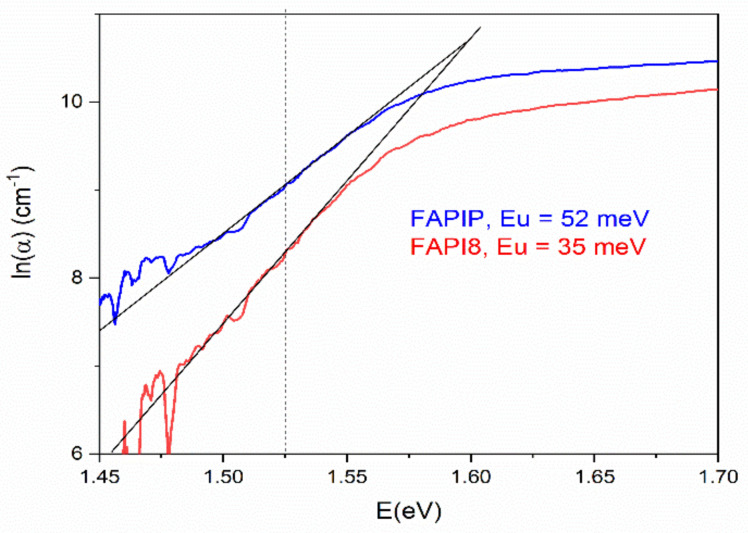
The absorption coefficient α versus photon energy *E* with the graphical estimation of the Urbach slope.

Figure 10 displays the spectral distribution of absorption coefficient in the log scale. The linear part of the distribution can be described by the relation [45]:

(3) $$\alpha =\alpha_{0}\times e^{\left[\frac{\left(E-E_{0}\right)}{E_{u}}\right]}$$

where *E_u_* is the Urbach energy while *α*_0_ and *E*_0_ are the characteristic values of the actual material. *E_u_* is defined as the measure of the density of states at the edge of valence and conductive bands related to defects in the material. Smaller *E_u_* values are expected for lesser defects in the material.

Generally, *E_u_* presents different types of disorder [46] which can be described as a sum of three contributions [47]:

(4) $$E_{u}=k_{0}\left(W_{T}^{2}+W_{X}^{2}+W_{C}^{2}\right)$$

where *k*_0_ is a constant while *W_T_*^2^, *W_X_*^2^, *W_C_*^2^ are mean-square deviations (MSD) from the electric potential of a perfectly ordered structure caused by temperature disordering, structural disordering, and compositional disordering, respectively. As summarized by Studenyak et al. [48], the temperature disordering (represented by *W_T_* MSD) is mainly caused by lattice thermal vibrations; structural disordering (*W_X_* MSD) can be intrinsic (e.g., vacancies or dislocations) or induced by external factors (e.g., ion implantation, dangling bonds, hydrogenation) while compositional disordering (*W_C_* MSD) is caused by the atomic substitution in mixed crystals.

Analogous to amorphous inorganic materials [49], in perovskite films, there is a linear dependence of *E_g_* on *E_u_* and smaller *E_u_* values result in larger *E_g_*, suggesting how the bandgap energy is affected by the degree of the disorder [50]. In our case, the blue-shift of *E_g_* and the position of the luminescent peak in PL experiments were observed. Specifically, smaller *E_u_* of FAPI samples with PVP comparing with pure FAPI samples implies better structural ordering, since a change of the basic chemical composition by polymer addition is unlikely. This conclusion strongly supports the model mentioned above in which polymer doping passivates the defects on the surface of crystals that built structure by elimination of dangling bonds, as was observed by other authors in the mixed cation [23] and the Cs cation [25] perovskite materials.

In conclusion, the UV-Vis results complement the PL findings and support the statement that the inclusion of PVP reduces the defects and consequentially has an effect on the density of states. A lesser amount of defects leads to fewer trap states at the band edges. By calculating the *E_u_* for both films, we can come to the conclusion that the PL findings of bandgap blue-shift further supports defect reduction by PVP inclusion inside the material.

### 3.4. Electrical Properties

**Figure 11:**
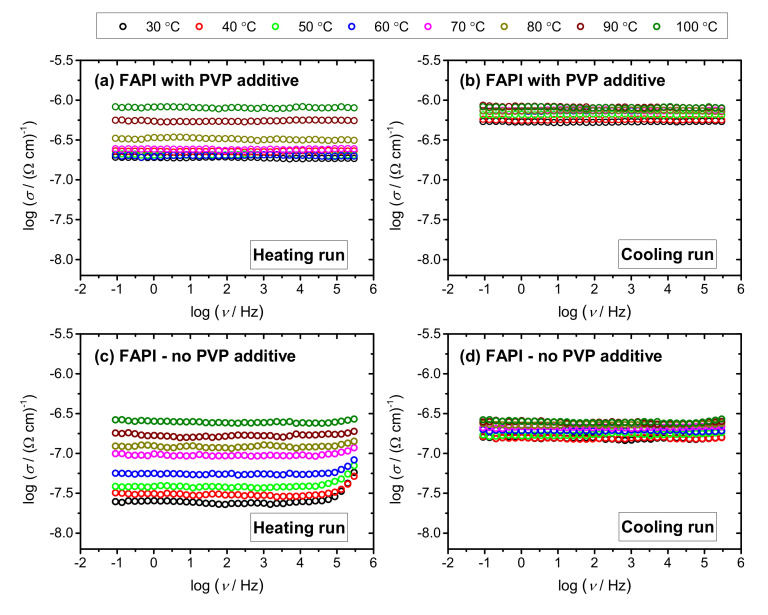
Conductivity spectra for FAPI perovskite thin films on ZNR/glass substrate with (**a**,**b**) and without (**c**,**d**) PVP additive in heating (**a**,**c**) and cooling (**b**,**d**) run.

Electrical conductivity spectra at different temperatures for FAPI thin films with and without PVP additive in heating and cooling run, are presented in Figure 11a–d. It is observed that both thin films show conductivity isotherms which are frequency-independent in a wide frequency range. The obtained plateaus correspond to DC conductivities and are typical for fast electronic transport. Moreover, it can be observed that the heating runs show a wider variation of conductivity with temperature, see Figure 11a,c, in comparison to cooling runs, see Figure 11b,d. In all cases, DC conductivity is thermally activated and increases with temperature showing semiconducting behavior.

**Figure 12:**
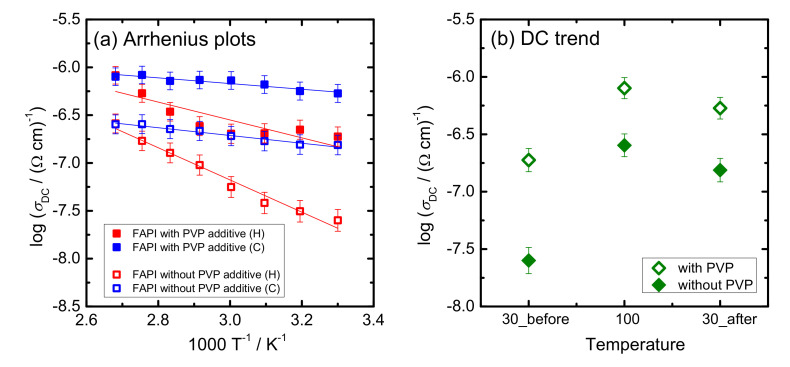
(**a**) Temperature dependence of DC conductivity (log(*σ*_DC_) vs. 1000/T) for FAPI perovskite thin film on ZNR/glass substrate with and without PVP additive and (**b**) DC-conductivity at 30 °C before heating, at 100 °C and after cooling at 30 °C (solid lines in Figure 12b). The observed increase in conductivity was one order of magnitude in the case of the film without the PVP additive and the half order of magnitude for the sample with the PVP additive. Second, in both heating and cooling runs, FAPI perovskite thin film with PVP additive shows higher electrical conductivity than its counterpart.

The DC conductivity exhibits Arrhenius temperature dependence and hence has characteristic activation energy (Figure 12a). The activation energy for DC conductivity, *E_DC_*, was determined from the slope of log(*σ_DC_*) vs. 1000/T using the equation:

(5) $$\sigma_{DC}=\sigma_{0}^{*}e^{\left[\frac{-E_{DC}}{k_{B}T}\right]}$$

where *σ_DC_* is DC conductivity, σ_0_^*^ is the pre-exponential factor, *E_DC_* is the activation energy, *k_B_* is the Boltzmann constant and *T* is the temperature (K). Values of *E_DC_* in heating and cooling run for both samples, FAPI perovskite thin film with and without PVP additive, are listed in Table 2 along with the values of DC conductivity at 30 °C. The values for DC conductivity at 30 °C were in the range 10^–7^–10^–8^ (Ω cm)^−1^, whereas the activation energy spans from 6 to 32 kJ/mol.

While the changes in the electrical properties of the film upon the addition of PVP are obvious, it is important to understand the physical origin of the influence of PVP additive on the electronic transport in ZnO nanostructures. The literature reports that nanostructured ZnO materials (with the preferred orientation) as ETL show a positive effect on electrical conductivity due to a decrease in the concentration of grain boundaries which usually have a blocking effect on the transfer of electrons. For instance, Wu et al. [51] showed that the adsorption sites on intergranular surfaces in ZnO nanowires lead to low conductivity. Oktik et al. [52] correlated a large density of extrinsic electron traps at grain boundaries, originating from oxygen chemisorption on ZnO films, to the presence of a barrier for charge-carrier conduction. A vacuum or reducing atmosphere desorbs the oxygen and results in a decrease in the grain boundary potential barrier and an increase in the effective charge-carrier concentration.

In our study, temperature-dependent impedance spectroscopy measurements were conducted in nitrogen atmosphere which resulted in different trends in DC conductivity in the heating/cooling runs (Figure 12 and Table 2). In a heating run, oxygen becomes desorbed from grain boundary sites and in the cooling run, a high electrical conductivity is retained along with significantly lower activation energy (6–8 kJ/mol vs. 18–32 kJ/mol, see Table 2). Such behavior is observed for both samples, FAPI with and without the addition of PVP polymer. The observed decrease in activation energy (heating vs. cooling) indicates facilitated charge transfer from the active FAPI perovskite layer to ETL in the cooling run. In particular, from the difference between heating and cooling runs for the two samples, it can be inferred that the influence of oxygen chemisorption is less pronounced in FAPI perovskite with PVP. This result can be related to a better FAPI incorporation with and between ZNR which decreases grain boundaries effect and improves stability and transport properties of ZnO/FAPI layer.

Moreover, perovskite thin films with PVP additive on ZNR/glass substrate exhibit higher electrical conductivity than their counterpart without PVP polymer in both runs (see Figure 12b). After the heating/cooling run, the observed difference decreases from one to half an order of magnitude, respectively. At the same time, the activation energy in the cooling run is within the same range (6–8 kJ/mol). Thus, it can be concluded that the process of better infiltration of FAPI perovskite with PVP into ZnO nanostructures has a positive impact on the charge transfer and along with improved structural stability, opens new routes for the development of material interesting in the context of PSC application.

**Figure 13:**
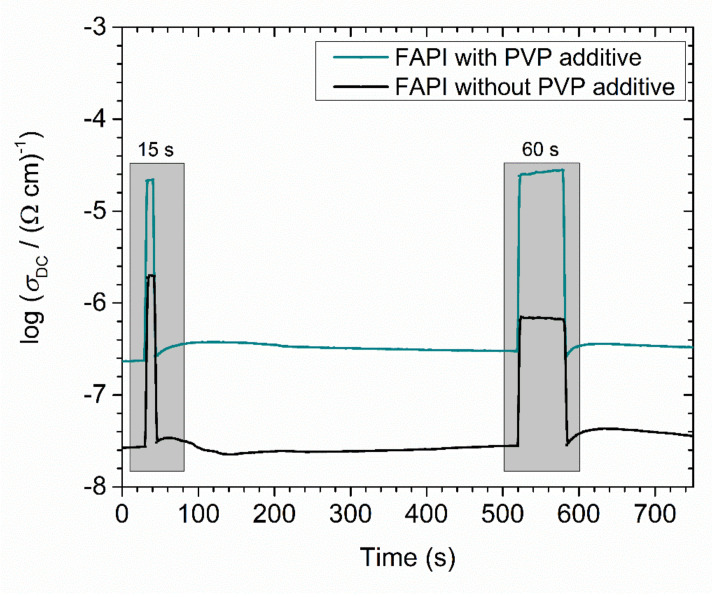
Photoconductivity measurements at 1 Hz after 15 and 60 s exposure to 1 Sun for FAPI perovskite thin film with and without PVP additive on ZNR/glass substrate.

In the next step, we investigated photoconductivity in both samples induced by illumination of 1 Sun. From Figure 13, it can be seen that the increase in photoconductivity upon exposure, as well as its decay after the illumination, is almost instant. The increase in photoconductivity is 1.5 and 2 orders of magnitude for FAPI thin film without and with PVP polymer, respectively.

Moreover, a closer look at Figure 13 reveals that during the second illumination, the increase in photoconductivity is slightly lower for the FAPI thin film without PVP polymer, whereas when PVP is added observed increase maintains the same extent. The observed effect can be attributed to the passivation of grain boundary defects and its impact on the hysteresis effects of perovskite polycrystalline thin films [53].

## 4. Conclusions

By inclusion of polyvinylpyrrolidone (8 mg/mL) into the FAPbI_3_ thin film, a suitable simple material for active layers in perovskite solar cells, we stabilized the cubic perovskite phase, which otherwise undergoes phase decomposition. FAPbI_3_ was deposited from a single solution on nanostructured ZnO using a one-step method with the inclusion of an anti-solvent step. ZnO nanostructures were prepared in the form of ZnO nanorods, as an appropriate candidate for low temperature processed electron transport layer in perovskite photovoltaic devices.

It was shown that the inclusion of PVP, as a stabilization additive in FAPbI_3_ polycrystalline thin films, facilitated infiltration of FAPI perovskite into ZnO nanostructures and stabilization of the active α-FAPbI_3_ phase for more than 2 months.

Measurements of optical properties show a blue shift in the optical gap, *E_g_*, and a luminescent peak by the addition of PVP. The intensity of photoluminescence of FAPbI_3_ thin films with PVP is much larger comparing with pure FAPbI_3_ which implies fewer non-radiative recombination centers and indicates lower defect density in the material with PVP. This hypothesis was further supported by the analysis of absorption coefficient close to *E_g_* that resulted in estimation of Urbach energy, *E_u_*. It was found that *E_u_* is lower for FAPbI_3_ thin films with PVP than for pure FAPbI_3_ which assumes better structural ordering of the former and explains the observed blue-shift of *E_g_* and photoluminescence peaks.

Electrical characterization of perovskite thin films on ZNR and glass showed Arrhenius temperature dependence with different trends in DC conductivity in heating/cooling runs. For both samples, FAPI with and without PVP, oxygen gets desorbed from grain boundary sites in heating, so in subsequent cooling high electrical conductivity is retained. The influence of atmosphere is found to be less pronounced in FAPI perovskite with PVP. This result supports the hypothesis that better incorporation of PVP with and between ZNR leads to better stability and transport properties of the active ZnO/FAPI layer. Furthermore, perovskite thin film with PVP additive shows higher electrical conductivity than its counterpart which again indicates that the infiltration of FAPI perovskite into ZnO nanostructures facilitates charge transfer in the ZnO/FAPI layer.

Comparison of both thin films upon exposure of 1 Sun shows a higher photoconductivity effect and better cycling properties for FAPI thin films with the added PVP polymer. This is a direct consequence of smaller amounts of defects in the material. These results further support the field of organometallic lead halide perovskites with the goal of promoting perovskite thin-film materials as the leading technology in 3rd generation PV devices.

## Figures and Tables

**Table 1:** Contact angle (CA) values for the solvent and polymer/solvent mixture.

Sample	CA(Glass)/(°)	CA(ZnO)/(°)
DMF/DMSO	32.73 ± 0.85	3.96 ± 0.38
PVP + DMF/DMSO	29.10 ± 1.02	1.62 ± 0.38

**Table 2:** The DC conductivity, *σ*_DC_, and activation energy, *E*_DC_ for FAPI thin films with and without PVP polymer on ZNR/glass substrate.

Sample	*σ_DC_*^a^/(Ω cm)^−1^ ±0.5%	*σ_DC_*^b^/(Ω cm)^−1^ ±0.5%	*E_DC,_*_Heating_/kJ mol^−1^ ±0.5%	*E_DC,_*_Cooling_/kJ mol^−1^ ±0.5%
With PVP	1.9 × 10^−7^	5.4×10^−7^	18	6
Without PVP	2.5×10^−8^	1.5×10^−7^	32	8

^a^ Value at 30 °C before heating/cooling run, ^b^ value at 30 °C after heating/cooling value.

## Data Availability

For data availability please send mail to vkojic@irb.hr.
